# The effect of adding TENS to stretch on improvement of ankle range of motion in inactive patients in intensive care units: a pilot trial

**DOI:** 10.1186/s13102-019-0129-5

**Published:** 2019-08-15

**Authors:** MohammadBagher Shamsi, Aliakbar Vaisi-Raygani, Asghar Rostami, Maryam Mirzaei

**Affiliations:** 1 School of Allied Medical Sciences, Kermanshah University of Medical Sciences, Kermanshah, Iran; 2 Department of Nursing, School of Nursing and Midwifery, Kermanshah University of Medical Sciences, Kermanshah, Iran; 3 Clinical Research Development Center, Imam Reza Hospital, Kermanshah University of Medical Sciences, Kermanshah, Iran

**Keywords:** Transcutaneous electrical nerve stimulation (TENS), Stretch, Range of motion, Intensive care unit

## Abstract

**Background:**

Patients hospitalized in intensive care units (ICUs) are susceptible to joint contracture and diminished range of motion. This is due to immobility as well as other underlying factors such as brain damage. Joint contracture causes functional disorders thereby diminishing the quality of life of patients following the intensive care period. Recent studies have introduced transcutaneous electrical nerve stimulation (TENS) as a new method for preventing and treating joint contracture. This study was performed to determine the effect of adding TENS to stretch on the range of ankle motion in patients hospitalized in ICUs.

**Methods:**

Thirty-six patients admitted to the ICU ward of the hospital who were not able to move their legs voluntarily were assigned randomly into experimental (*n* = 18) and control (*n* = 18) groups. The intervention group received TENS along with manual stretch in the ankle three times a week for 2 weeks. The control group only received stretch in the ankle for the same time. The extent of dorsiflexion and plantar flexion of the ankle was measured using a standard goniometer. Both groups were evaluated before and one and 2 weeks after the intervention. The obtained data were analyzed by SPSS 21 through analysis of covariance and repeated measures ANOVA tests.

**Results:**

In both groups, the increase in the ankle range of motion parameters was significant over time (means ranged over 44–48 for plantar flexion and means ranged over 5–11 for dorsiflexion, *P* < 0.001 for all of time points). The increase in ankle plantar and dorsiflexion in experimental group was significantly more than control group (mean between-group differences ranged over 1.35–3.57 within 95% CI of 1.04 to 4.01, *P* < 0.001).

**Conclusion:**

Adding TENS to stretch may provide more improvement in ankle dorsiflexion and plantar flexion.

**Trial registration:**

Trial registration: This study was registered in the Iranian Clinical Trial Center with the code IRCT2017010814333N64, registered 20 January 2017.

## Background

The developments in the treatment and management of patients in intensive care units (ICUs) have enhanced the chances of survival of these patients. However, as a result of survival, the probability of incidence of various complications in these patients increases during the hospitalization period [1]. During this period, due to the patients’ immobility, some problems occur in the natural physiology of the body. If they are not prevented, they can result in the incidence of irrecoverable consequences [2]. One important complication of long immobility in these units is joint contracture. The main characteristic of this is the diminished range of motion in patients [3, 4]. Long-term immobility plays the main role in the development of this problem [3, 5]. Contracture usually occurs in people with joint problems or stroke patients along with the elderly or immobile patients [6, 7]. Patients hospitalized in ICUs are more susceptible to joint contracture due to immobility as well as other underlying factors such as cerebral damage [3, 5].

Although stretching and passive motions are often performed by nurses or physiotherapists for patients in ICUs, incidence of joint contracture is reported. Hence, more measures should be taken to prevent contracture [3, 5, 8].

Detection of the extent of contracture of joints is based on the measured range of motion. In a contracture state, range of motion is restricted. With respect to the ankle, the range of motion is measured from the maximum plantar flexion angle until the maximum dorsiflexion angle of the joint [3, 8]. If physiotherapy is provided to patients in the initial days spent in the ICUs as a preventive, it will result in a significant improvement in their functional state in the post-treatment period [3, 5, 6, 8]. The first week of rehabilitation measures is considered as a critical period, as these measures are associated with 13% reduction in atrophy of quadriceps, leg, and foot muscles [3, 5]. These interventions can also have long-term effects [9].

This initial prevention reduces therapeutic costs and prevents invasive measures such as surgery. Various therapeutic measures are taken to prevent and treat ankle contracture, based on the situation of every person and the severity of the contracture [10, 11]. These include active and passive stretch, use of cast, splint, intramuscular Botox injection, and surgery to release the tendon to prevent contracture of the joint. Active and passive stretching are among the most common major measures for preventing and treating ankle contracture [12]. Today, stretching is used to improve the flexibility of muscles and tendons. Recent studies on humans have shown the effectiveness of rehabilitation measures in enhancing the physical and functional ability of patients [5, 8, 13].

Further, in recent studies, the effect of transcutaneous electrical nerve stimulation (TENS) on improving the range of motion of joints, contracture, and spasticity has been reported [8, 13]. TENS is a simple and safe non-invasive method for mitigating pain, which is widely used in therapeutic clinics. Its uses include mitigating acute and chronic pain, anti-nausea effects, and increasing blood circulation [14]. Thus, the aim of this study is to investigate the effect of adding TENS to stretch (which is a common method in preventing and treating joint contracture) in preventing the diminished range of ankle motion in patients admitted to the ICU.

## Methods

### Design

This study was performed as a double blind controlled clinical trial with parallel groups on patients admitted to the ICUs of academic-healthcare hospitals affiliated to the Kermanshah University of Medical Sciences in 2016. This study was approved by the ethics committee of this university and registered in the Iranian Clinical Trial Center with the code IRCT2017010814333N64.

### Participants

The inclusion criteria included hospitalization in the ICU ward for more than 1 week, age between 18 and 60 years, patients who were not able to move their legs voluntarily (unconscious patients and those who were not able to move their lower limbs due to paralysis), agreement to sign the written consent form by the patient or his or her guardian (in case of unconsciousness), lack of disease or musculoskeletal disorder according to a physical examination, lack of damage inside or around the ankle, no history of treatment by electrical stimulation in the ankle zone, no skin disease hindering placement of electrodes or causing sensitivity to electrical stimulation, absence of electrical implant devices (such as pacemaker), and lack of sever spasticity that prevented joint movement. However, the exclusion criteria included skin sensitivity to stimulating electrodes, TENS intolerance and acquiring the ability to move the lower limbs voluntarily during the interventions.

The patients were chosen using the convenience sampling method. Thereafter, they were assigned into two groups (experimental and control groups) with a block size of two through a permuted block random sampling method by a statistical expert not involved in data collection (allocation concealment). The sample size was estimated to be at least 18 patents in each group according to a study by Oo [15] considering the mean ± SD for composite spasticity score as the key variable, confidence interval of 95% and power of 80%. At the beginning of the study, the objective and method of study implementation were explained to the patients or their companions. Also, written consent was taken from them and then the demographic information (age and gender) questionnaire was completed with the help of information from the patients’ file.

### Outcome measures

The first outcome measurement in this study was passive ankle range of motion along the plantar flexion and dorsiflexion directions. The ankle’s range of motions was measured using an international manual goniometer device. Compared to radiography, the measurement of a joint angle with the goniometer has a high level of accuracy [16]. It has been claimed that measuring a joint angle with a universal goniometer has moderate to excellent reliability [17]. Therefore, it can be used as a repeatable instrument for measuring the range of motion of human joints.

**Fig. 1:**
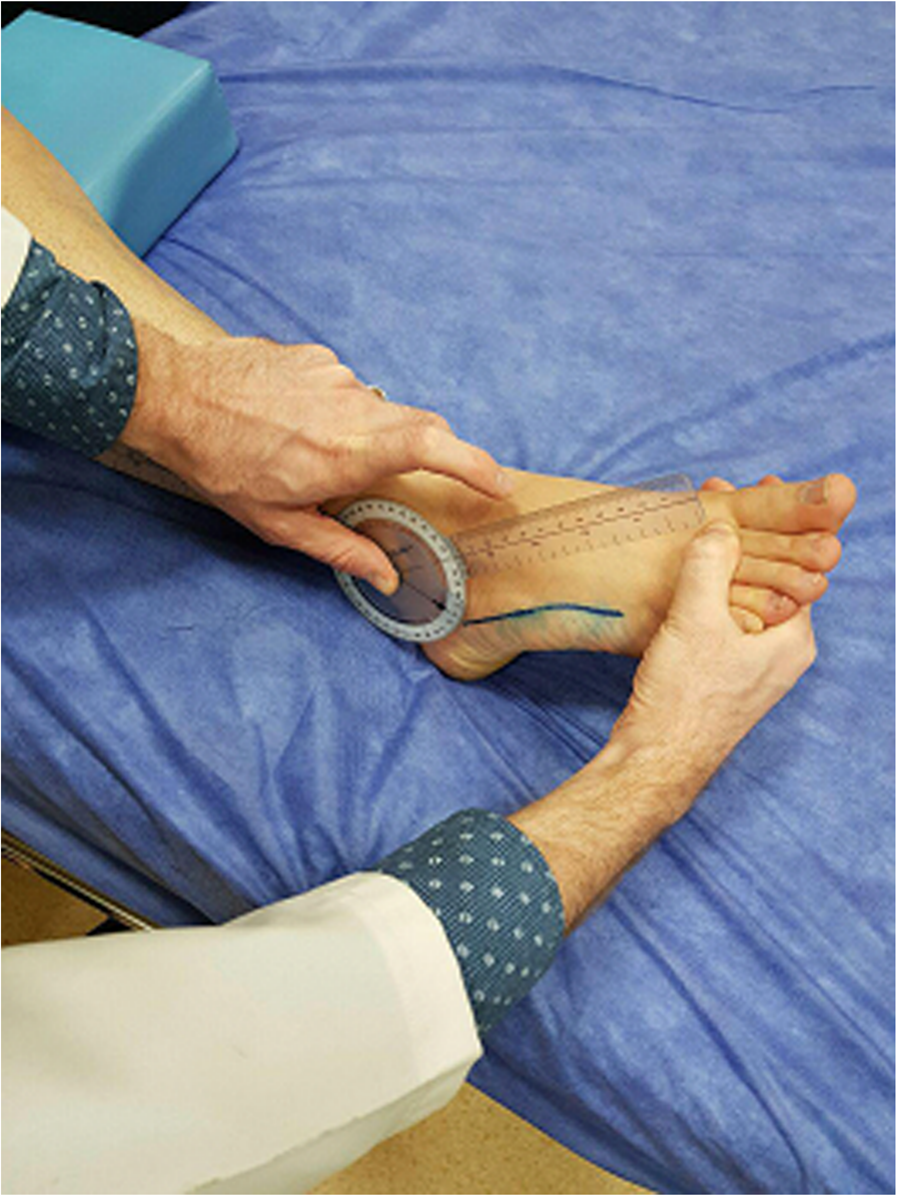
Ankle range of motion measurement

To measure the range of motions, the patient was placed in a supine position, with absolutely no deviation to right or left in the hip joint (Fig. 1). The measurement technique is described in similar studies [18].

Ankle joint ROM measurement was done bilaterally and the mean value was considered for calculation. One independent assessor who was not the person who applied the stretches, travelled to all three sites and did all ankle measurements.

### Interventions

**Fig. 2:**
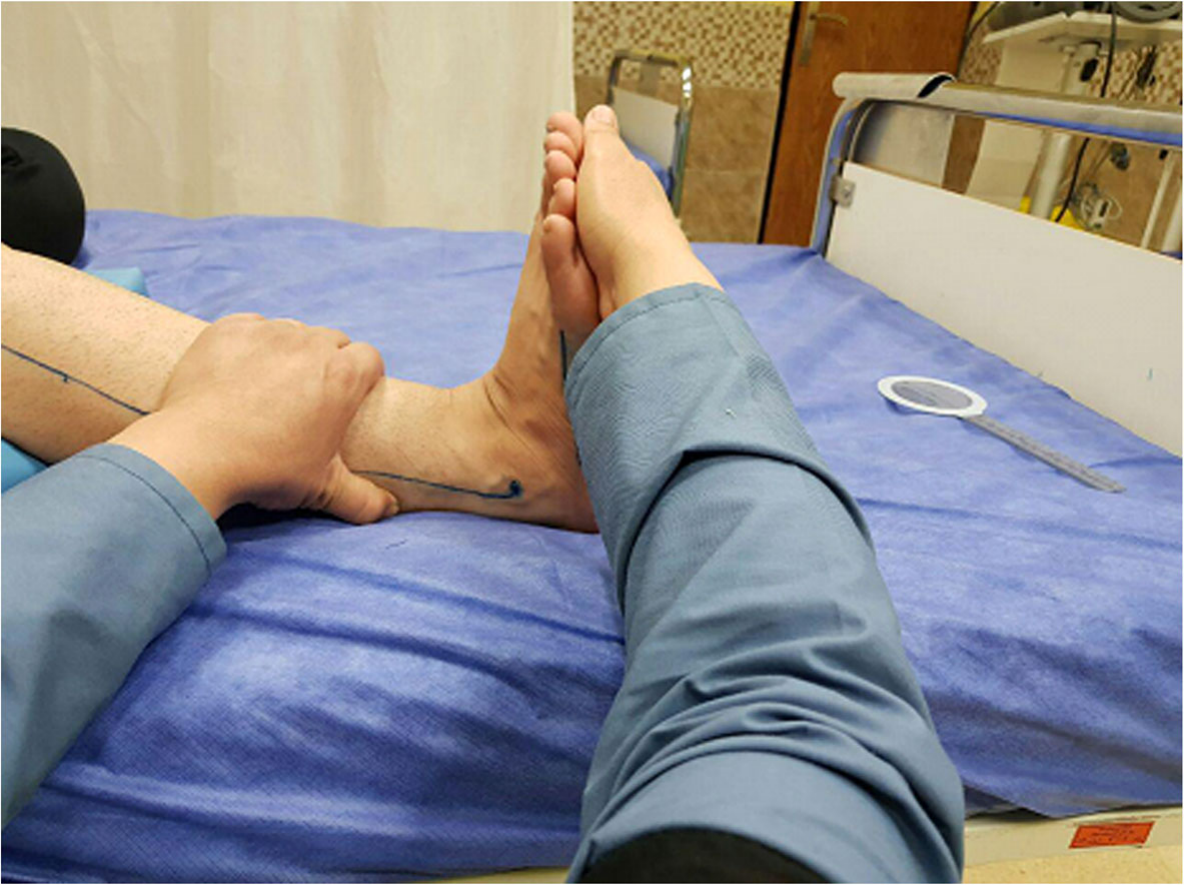
Ankle stretching

In the control group, when the patient was in supine position with a straight knee, the therapist dorsiflexed the patient’s ankle by applying gentle pressure. Keeping the exertion of the same force but with two degrees less of dorsiflexion, he/she maintained the stretching (Fig. 2) [19]. The stretch was performed for 10 min in each session. For this purpose, there were five 2-min stretches with 1 min of rest between every two stretches. A therapist who had been trained by an experienced physiotherapist applied the ankle stretches for all patients. He tried to keep the applying force equal for different joints.

In the experimental group (TENS along with stretch), before performing the stretching, TENS was used. First, the skin of the ankle region was cleaned using cotton smeared with 70% alcohol to decrease the skin resistance. Next, two electrodes (4*4 cm) were placed on both sides of the ankles. Finally, the TENS stimulations were applied for 30 min at a frequency of 100 Hz, duration of 0.2 ms, and intensity of 15 mA [15, 20, 21]. The electrical stimulation was applied using the ES-320 stimulator (ITO, Japan). Among the TENS mechanism is the activation of nerve impulse in a large number of alpha afferent nerve roots, resulting in the excitation of inhibitory neurons of the dorsal horn or the release of endorphin or both [14]. Further, TENS enhances blood circulation close to the electrodes, which indirectly contributes to regeneration, reduction of spasm and contracture, and relaxation of muscles [14]. The TENS mechanism in mitigating spasticity and contracture of ankle can be due to the excitation of afferent fibers in the peroneal nerve [15].

Increased blood circulation close to the electrodes is also effective in reducing ankle contracture. As there is no need for active participation by the patient in TENS application, it can be used by people who have not been able to perform physical activity for a long time. They can benefit from TENS thanks to its safety and low complications as well as convenience [22].

The interventions of both groups were done three times a week for 2 weeks. The therapist who performed the interventions were not informed about the participants groups. In the first session, the interventions (ankle stretching or TENS stimulation) were explained for patients (or their relatives in case of unawareness).

### Data analysis

Data analyses were conducted by SPSS 21.0 (SPSS Inc., Chicago, IL) and *P* values of < 0.05 were set at level of significance. Frequency (percent) and mean (standard deviation) were used to summarize the accumulated data. The comparisons of background variables including demographic variables and clinical parameters were investigated between the experimental and control groups using the t-test and the chi-squared test.

Normality was confirmed for all ankle range of motion parameters by the Kolmogorov–Smirnov one-sample test. The within-group differences in time trend for each group was assessed by repeated measures analysis of variance. For this test, assumption of the sphericity (consistency of correlations) was not confirmed by Mauchly’s test. Therefore, *P* values were stated based on the Greenhouse–Geisser test in both groups.

Additionally, the basic measurements of the ankle range of motion parameters were adjusted as covariate and differences between groups from baseline to weeks 1 and 2 were assessed by analysis of covariance (ANCOVA).

## Results

**Fig. 3:**
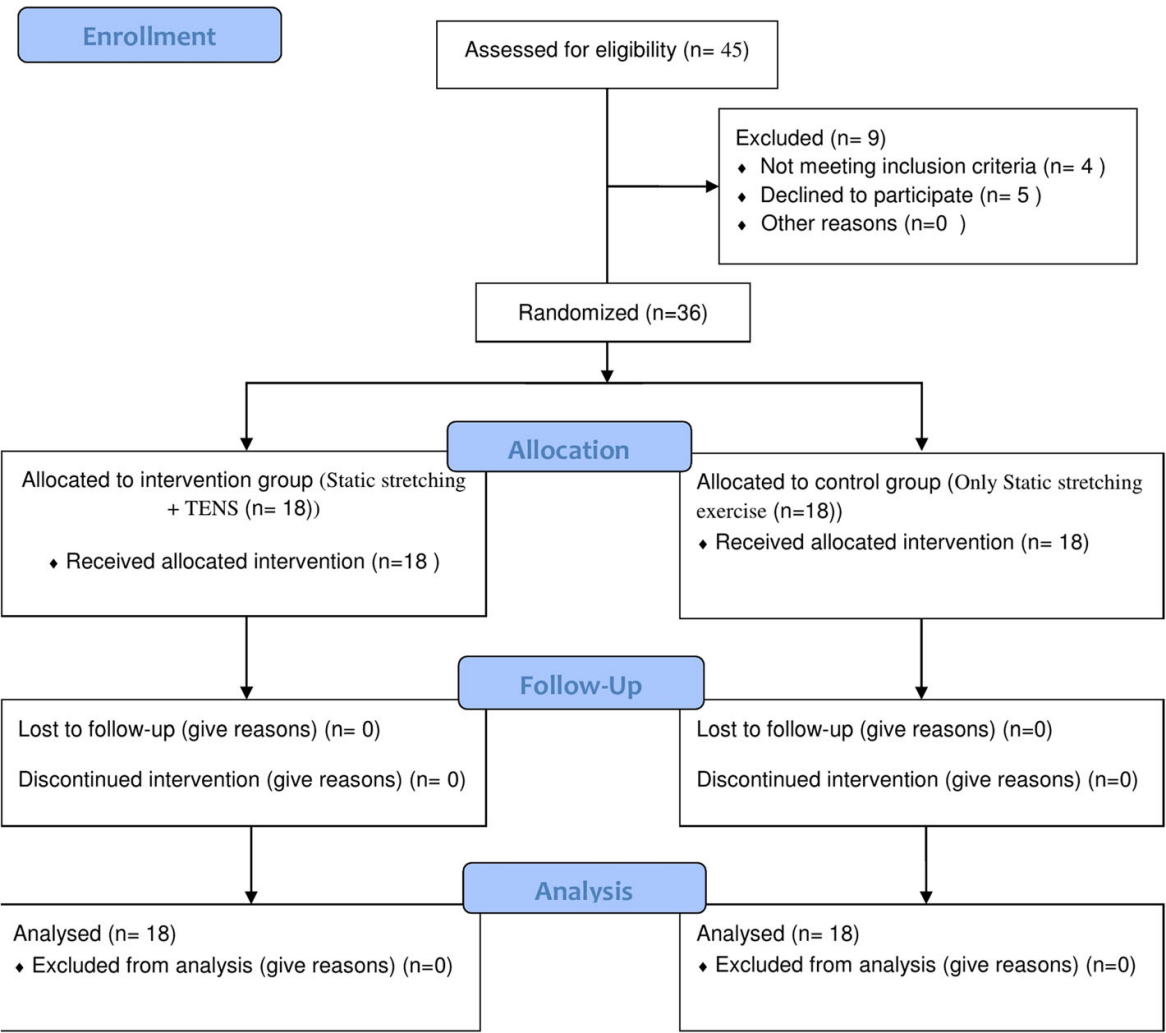
Flow diagram of study selection and data collection process

In this trial, all the patients remained in the study and there was no loss to follow up on during the study (Fig. 3). The comparison of demographic variables of patients showed that there was no significant difference between the groups in terms of distribution of sex, history of underlying illness and drug use, cause of hospitalization, as well as mean age and level of consciousness (in all cases, *P* > 0.05; Table 1).

**Table 1:** Demographic information of the groups

Group	Experimental group (*n* = 18)	Control group (*n* = 18)	*p*-value
Age(year)	50.66(11.60)	51.83(7.94)	0.727^a^
Level of consciousness (Based on Glasgow Coma Scale)	7.01(0.68)	7.34(0.84)	0.098^a^
Cause of hospitalization			
Brain hemorrhage	6(33.33)	7(38.88)	0.143^b^
Stroke	7(38.88)	1(5.55)	
Trauma	2(11.11)	5(27.80)	
Cancer	1(5.6)	3(16.66)	
Respiratory disease	2(11.11)	2(11.11)	
Use of anesthetic and muscle relaxant			
Yes	9(50)	6(33.33)	0.310^b^
No	9(50)	12(66.67)	
Sex			
Female	5 (27.78)	7 (38.89)	0.480^b^
Male	13 (72.22)	11 (61.11)	
Background or underlying disease			0. 901^b^
High Blood Pressure	7(38.9)	8(44.4)	
Ischemic heart disease	1(5.55)	0(0)	
Cancer	3(16.71)	2(11.1)	
Diabetes	2(11.21)	2(11.11)	
Respiratory disease	1(5.6)	2(11.1)	
No history of obvious diseases	4(22.21)	4(22.22)	

Data are number (percent) except age and level of consciousness that presented as means (SD))/ ^**a**^Based on t independent test/ ^b^Based on chi-square test

There was no statistical difference between groups regarding drug use.

**Fig. 4:**
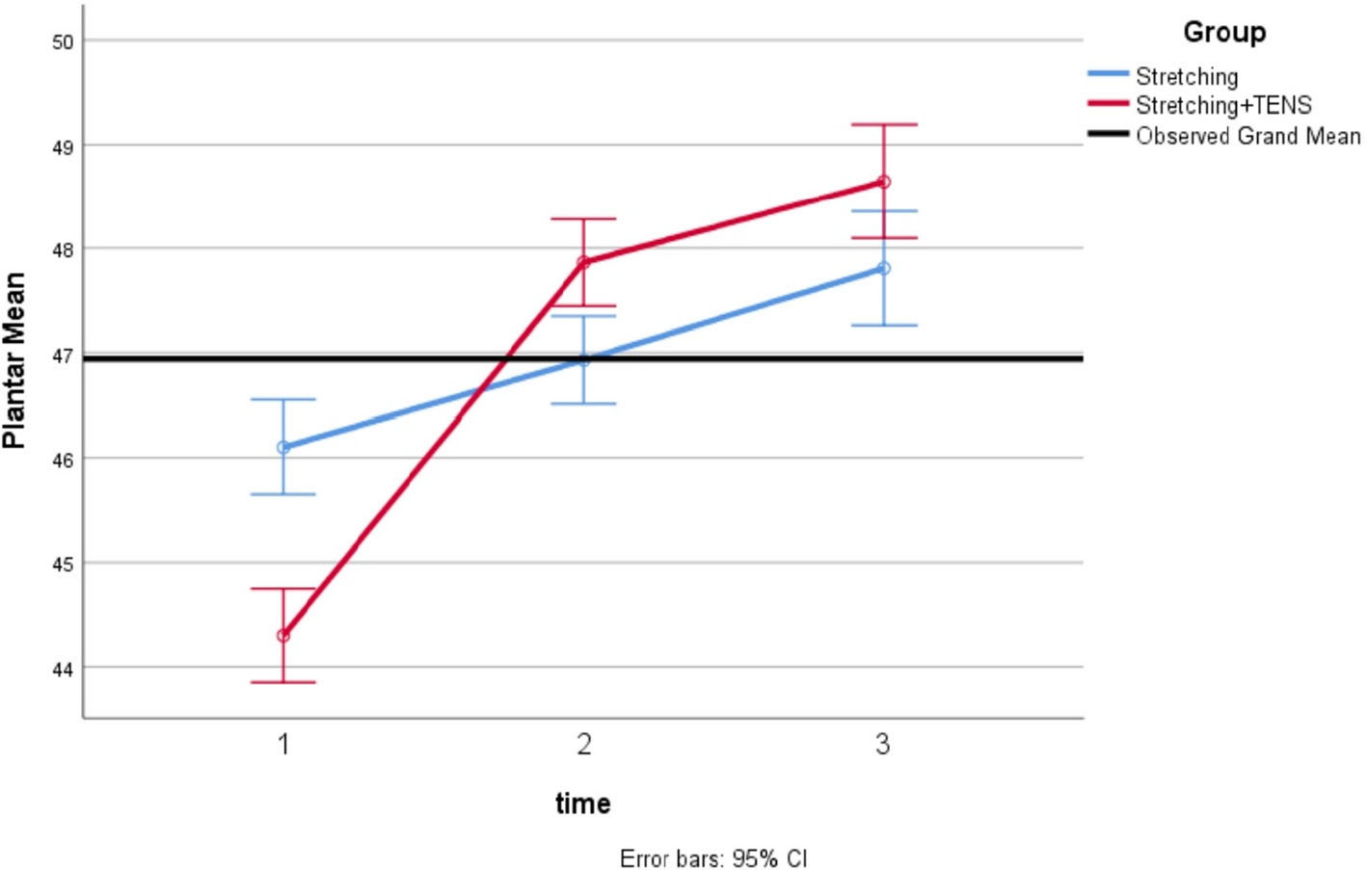
Mean of ankle plantar flexion in the two groups

**Fig. 5:**
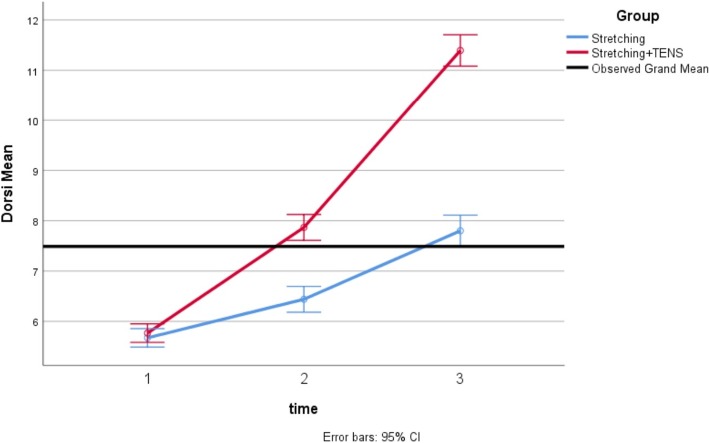
Mean of ankle dorsiflexion in the two groups

Mean values of dorsiflexion and plantar flexion during the study are shown in Figs. 4 and 5. In both groups, the increase in the mean values of ankle range of motion parameters was significant over time. Based on the Sidak post-hoc test, the differences between the first and the second measurements, between the second and the third measurements, as well as between the first and the third measurements were significant (means ranged over 44–48 for plantar flexion and means ranged over 5–11 for dorsiflexion, *P* < 0.001 for all of time points; Table 2).

**Table 2:** Repeated measures ANOVA of mean and standard deviation related to ankle range of motion parameters scores in three measurements for each group

Ankle motion	Time of measurement period	Experimental group (*n* = 18)^*^	Control group (*n* = 18)^*^
Plantar flexion	Baseline	44.30 (1.16)	46.10(0.64)
	1 week after baseline	47.86(1.02)	46.93(0.68)
	2 week after baseline	48.46(1.38)	47.81(0.84)
	* Repeated measures ANOVA (inter-group)	*F* = 234.42; df = 2; *P* < 0.001	*F* = 198.84; df = 2; *P* < 0.001
Dorsiflexion	Baseline	5.77(0.31)	5.67(0.44)
	1 week after baseline	7.87(0.56)	6.44(0.51)
	2 week after baseline	11.39(0.57)	7.80(0.71)
	^#^Repeated measures ANOVA (inter-group)	*F* = 103.52; df = 2; *P* < 0.001	*F* = 105.55; df = 2; *P* < 0.001

^*^Mean (SD) was reported/ ^#^ *P* value from Greenhouse-Geisser test has been reported based on the results of Mauchly’s test

According to the results of the effect of the experimental group compared to the control group on the variables, there were statistically significant differences between the groups with respect to plantar flexion and dorsiflexion change at the end of the study (Table 3). These differences in values of the ankle range of motion existed in the first and second measurements (mean between-group differences ranged over 1.35–2.5, (95% CI = 1.04 to 2.70), *P* < 0.001). After making adjustments for the baseline measurement, the differences between the experimental group and the control group regarding change in ankle plantar and dorsiflexion from the first measurement (before intervention) and the last measurement (after intervention) remained significant (mean between-group differences ranged over 2.67–3.57, (95% CI = 2.04 to 4. 01), *P* < 0.001).

**Table 3:** Comparison of changes in ankle range of motion parameters among the 2 groups of study

Ankle motion	Difference between stages of measurement	Mean Between-group difference^*^	95% CI for difference^*^	^#^*P* value (between groups)
Plantar flexion	Second and first	2.27	(1.76 to 2.77)	0.001
	Third and first	2.67	(2.05 to 3.28)	0.001
Dorsiflexion	Second and first	1.35	(1.04 to 1.65)	0.001
	Third and first	3.57	(3.12 to 4.01)	0.001

*Mean between-group difference and 95% CI for difference were reported/ ^#^*P* value is reported based on the analysis of covariance

## Discussion

The aim of this randomized controlled trial was to compare the effectiveness of adding TENS to a passive range of motion stretch in hospitalized patients in ICUs to that of passive stretch alone on ankle contracture. After 2 weeks of treatment, the between-group comparisons showed that ankle dorsiflexion and plantar flexion improved more in the TENS and stretch group than in the merely stretch group.

Limited mobility and increased risk of falls, which are associated with older adults and many neurological conditions, may be related to deficits of strength, joint position sense and balance control. Applying TENS at the foot and ankle has the potential to be a highly beneficial intervention in deficits in these abilities [23]. It is hypothesized that using TENS in the foot and ankle area would improve the balance and postural control [23]. As TENS improves strength, joint position sense and balance control, it is reasonable that it has effects on joint mobility.

To our knowledge, none of studies in the literature applied TENS at the foot and ankle for prevention or treatment of joint stiffness. However, there are many related studies on other joints. Therefore, the novelty of our study is investigating the effect of TENS on ankle stiffness in immobility conditions.

It has been shown in many studies that adding TENS to an intervention of joint movement (passive or active) had an impact on joint mobility improvement. Angulo et al. [24] studied patients undergoing total knee replacement (TKR) surgery. In the three days after the surgery, he used TENS along with a continuous passive motion (CPM) device, but he did not observe any significant difference in adding TENS to joint passive motion for enhancing the range of motion. In terms of the interventions, this study was similar to ours, but it was different in terms of the studied joint and hospitalization of the patients in the ICU. Unfortunately, no study investigating the effect of adding TENS to the joint motions in patients lacking consciousness in the ICU was found in the literature. Most of related studies were on knee joint as the involvement of this joint is more than others. In similar papers about other joints, as mentioned previously, different controversial results were observed.

The effect of TENS in mitigating pain is evident and widely accepted. Definitely, diminished pain in the joint will develop conditions which facilitate the joint motion, thus decreasing motion constraints.

Sometimes the limited range of motion of a joint is due to muscular spasm and the constraint resulting from the contraction of muscles. In such cases, the use of TENS and other neural stimulations will lead to resolution of the spasm and freedom of motion. Probably, in studies emphasizing the effect of TENS on reducing the limited motion, the origin of the abnormal contraction restraint has been muscular. In contrast, sometimes the limitation is due to soft tissue contracture and reduced flexibility of non-contractile soft tissues as well as joint stiffness and capsular adhesion. In these cases, electrical stimulations mitigate the pain temporarily and have no effect on resolving the joint limitations in the long term. Possibly, the studies implying the lack of effect of TENS in resolving joint constraint may belong to this group.

On the other hand, patients admitted to ICU wards experience pain in joints and most parts of the body due to immobility and long hospitalization. To avoid the pain developed in response to movement, the patient prefers to remain immobile. This vicious cycle causes further reduction in the range of motion of joints. By breaking this cycle and developing the opportunity whereby joints move without pain, use of TENS and reduction of pain may have been able to develop conditions to minimize the limited range of motion to an acceptable level.

Our study had some limitations: 1- Samples were recruited only from three hospitals and it was not possible to participate patients from more ones because this kind of patients was not admitted in other hospitals, 2- The frequency of interventions in both groups (three times a week) was low. If possible, applying the interventions every day may have more impact on variables. 3- Although there were no significant differences between groups in baseline data, the study groups are not quite balanced in respect to the cause of admission to ICU, due to the relatively small sample size. 4- Patients had different trial starting points, as it is feasible that any patient who had a prolonged stay prior to recruitment may have had a measurable limitation of ROM before treatment started.

## Conclusion

Adding TENS to stretch may provide more improvement in ankle dorsiflexion and plantar flexion in prevention of ankle joint stiffness. Thus, it can be recommended as a non-pharmacological treatment to manage the patients hospitalized in ICUs.

## Data Availability

The datasets used and/or analyses during the current study are available from the corresponding author on reasonable request.

## Abbreviations

ANCOVA: Analysis of Covariance
CPM: Continuous passive motion
ICUs: Intensive Care Units
TENS: Transcutaneous Electrical Nerve Stimulation
TKR: Total Knee Replacement
WOMAC: Western Ontario and McMaster Universities Osteoarthritis Index
